# Development of chloroplast microsatellite markers for *Glyptostrobus pensilis* (Cupressaceae)

**DOI:** 10.1002/aps3.11277

**Published:** 2019-07-17

**Authors:** Ya‐Dan Yan, Xin‐Yu Li, James R. P. Worth, Xue‐Ying Lin, Markus Ruhsam, Lu Chen, Xing‐Tong Wu, Min‐qiu Wang, Philip I. Thomas, Ya‐Feng Wen

**Affiliations:** ^1^ Central South University of Forestry and Technology Changsha Hunan 410004 People's Republic of China; ^2^ Forestry and Forest Products Research Institute Tsukuba Ibaraki 305‐8687 Japan; ^3^ Royal Botanic Garden Edinburgh 20A Inverleith Row Edinburgh EH3 5LR United Kingdom

**Keywords:** chloroplast microsatellite (cpSSR), Cupressaceae, *Glyptostrobus pensilis*, haplotypes

## Abstract

**Premise:**

*Glyptostrobus pensilis* (Cupressaceae) is a critically endangered conifer native to China, Laos, and Vietnam, with only a few populations remaining in the wild.

**Methods and Results:**

Using a complete chloroplast genome sequence, we designed 70 cpSSR loci and tested them for amplification success and polymorphism in 16 samples. Ten loci were found to be polymorphic and their genetic diversity was characterized using a total of 83 individuals from three populations in China. A total of 43 haplotypes were present, the effective number of haplotypes varied from 4.55 to 13.36, and the haplotypic richness ranged from 8.04 to 16.00. Gene diversity ranged from 0.81 to 0.97 (average 0.89). The number of alleles per locus and population ranged from one to eight, and the effective number of alleles ranged from 1.00 to 3.90. All polymorphic loci were successfully amplified in the related species *Cryptomeria japonica* var. *sinensis*,* Taxodium distichum*,* T. ascendens*, and *Cunninghamia lanceolata*.

**Conclusions:**

These newly developed chloroplast microsatellites will be useful for population genetic and phylogeographic analyses of *G. pensilis* and related species.

*Glyptostrobus pensilis* (Staunton ex D. Don) K. Koch, the only extant species in the genus *Glyptostrobus* Endl., is a relict conifer in the family Cupressaceae (Hao et al., 2016). In China, it is mainly distributed in the Pearl River delta region of Guangdong Province, the central region of Fujian Province, the lower reaches of the Minjiang River, and northeastern Jiangxi Province (Li and Xia, 2004). A few wild populations have recently been found in Laos and Vietnam, extending its latitudinal distribution from 28°N to 13°N (Averyanov et al., 2009; Thomas and LePage, 2011). The species preferred habitat of riverbanks and flood plains have been severely degraded by human activities (e.g., agriculture and rice cultivation) in many locations, which has led to a rapid decline of most *G. pensilis* populations (Li and Xia, 2004, 2005; Nguyen et al., 2013). Currently, the International Union for the Conservation of Nature (IUCN) Red List of Threatened Species has evaluated *G. pensilis* as Critically Endangered (CR) (Thomas et al., 2011).

Chloroplast microsatellites have been widely used to investigate the population genetic structure and phylogeographic history of a range of tree species (Ruhsam et al., 2016; Gryta et al., 2017). Previous molecular studies of *G. pensilis* have only used nuclear markers such as inter‐simple sequence repeats (Li and Xia, 2005; Wu, 2011), and recently Wang et al. (2019) developed 10 polymorphic nuclear microsatellite markers for this species. Compared with nuclear simple sequence repeats (SSRs), chloroplast SSRs (cpSSRs) are more likely to detect historical bottlenecks or genetic drift due to their uniparental inheritance, slower mutation rate, and lack of recombination (Ennos et al., 1999; Pleines et al., 2009; Li and Liu, 2012). Nguyen et al. (2013) analyzed *G. pensilis* populations from Vietnam using six cpSSRs; however, these loci were developed from *Pinus thunbergii* Parl. and designed for use in Pinaceae species (Vendramin et al., 1996). In this study, we developed new species‐specific chloroplast microsatellite loci using the complete chloroplast genome of *G. pensilis* (Hao et al., 2016). Additionally, we tested the transferability of these loci in four related species: *Cryptomeria japonica* (Thunb. ex L. f.) D. Don var. *sinensis* Miq., *Taxodium distichum* (L.) Rich., *T. ascendens* Brongn., and *Cunninghamia lanceolata* (Lamb.) Hook.

## METHODS AND RESULTS

We searched the complete chloroplast genome of *G. pensilis* (Hao et al., 2016; GenBank accession number KU_302768) for microsatellite loci exhibiting a minimum of eight repeats as these loci are likely to exhibit a higher level of polymorphism (Ueno et al., 2012). For loci with a minimum of eight repeats, primers were designed using the online software Primer3Plus (Untergasser et al., 2007) using default parameters. In total, 70 cpSSR loci were selected and evaluated for their amplification efficiency and level of polymorphism using 16 *G. pensilis* DNA samples from different populations (Appendix 1). DNA was extracted from *G. pensilis* leaves using a modification of the cetyltrimethylammonium bromide (CTAB) method (Tsumura et al., 1995).

PCR amplification was carried out in volumes of 15 μL using the following protocol: 7.5 μL of 2× Taq PCR Master Mix (Tiangen, Beijing, China), 0.75 μL of forward primer (10 μM), 0.75 μL of reverse primer (10 μM), 3 μL of 20–50 ng/μL DNA template, and 3 μL of ddH_2_O. The mixture was then cycled using the following profile: 94°C for 4 min; 34 cycles of 94°C for 30 s, 55°C for 30 s, and 72°C for 30 s; with a final extension at 72°C for 30 min. PCR products were visualized on a 1.6% agarose gel. All loci that could be amplified successfully were tested individually using 16 *G. pensilis* samples to establish their polymorphism. These amplifications were carried out using fluorescently labeled primers (FAM, HEX, TAMRA, and ROX; Applied Biosystems, Foster City, California, USA) and the same PCR protocol as detailed above. PCR products were run on an ABI 3730xL DNA Analyzer adding a GeneScan 500 LIZ internal size standard (Applied Biosystems) to size fragments. The software GeneMarker version 1.9 was used to score the electropherograms of all samples (Hulce et al., 2011). Sixty‐five of the 70 cpSSR loci amplified successfully across the 16 test individuals, but 55 loci were monomorphic, and only 10 loci were polymorphic (Table 1, Appendix 2). These polymorphic loci were used to investigate the genetic diversity of 83 individuals across three Chinese *G. pensilis* populations (Appendix 1).

**Table 1:** Characterization of 10 polymorphic chloroplast microsatellite loci developed in *Glyptostrobus pensilis*.^a^

Locus	Primer sequences (5′–3′)	Location	Repeat motif	Allele size range (bp)	GenBank accession no.
Gp_cp_1	F:(ROX)TGACACACGGGTCTGTATCA	ycf4 to psaI	(AT)_10_	261–271	MK386658
	R: GCCTTTGGTGGGCTTGTTTT				
Gp_cp_6	F:(FAM)GCTGTTCCCCTGTGCATCAT	trnL to trnF	(AT)_11_	195–203	MK386659
	R: GATCAATTTGTGTCTGCTTCTGT				
Gp_cp_7	F:(HEX)ACCTGTCTCAAATCGACTTCCC	ycf3 to psaA	(T)_11_	149–167	MK386660
	R: CTCCTCTTTCCAGACGAGACA				
Gp_cp_8	F:(HEX)TGAACCGATGACTTACGCCT	psb to trnE	(TA)_9_	267–279	MK386661
	R: AAATCGAATCCCCGTTGCCT				
Gp_cp_11	F:(TAMRA)AATCCTGAAAGTCGACTAGAATTAAGT	chlB to rps16	(AT)_23_	363–414	MK386662
	R: GCTAAGAGCATCTTCGAATAAAAATAG				
Gp_cp_12	F:(TAMRA)TTAAGTCGAGTGAGTCAGATGG	accD to clpP	(T)_11_	379–437	MK386663
	R: TGCCCATAGGATGCCAAGTG				
Gp_cp_13	F:(TAMRA)TGGGGGATCAAAATAACACAGA	rbcL to accD	(AT)_16_	208–334	MK386664
	R: GTTTTCCAATGTGAATTTGAAAATCGA				
Gp_cp_14	F:(FAM)TCCCCGCAGAACTATCGTTT	ccsA to petA	(T)_12_	208–214	MK386665
	R: AGGAAAGAATTTGGTAATCTTGGCT				
Gp_cp_17	F:(FAM)ACCTACCCAGAATTAGCAAGCC	trnD to psbM	(T)_12_	116–119	MK386666
	R: AGAATTGGCGGTTGCTTCCT				
Gp_cp_35	F:(HEX)TTTTCCTCTACCGCGAACCC	psaJ to rpl33	(A)_10_	115–117	MK386667
	R: ACTTCACCCCTCCTTGAATTCT				

a Optimal annealing temperature was 55°C for all loci.

The software Haplotype Analysis version 1.05 (Eliades and Eliades, 2009) was used to calculate the following statistics: number of haplotypes (*A*), number of private haplotypes (*P*), effective number of haplotypes (*N*_e_), haplotypic richness (*R*_h_), and gene diversity (*H*_e_). The software GenAlEx6.5 (Peakall and Smouse, 2012) was used to calculate the following parameters: number of alleles (*N*_a_), effective number of alleles (*N*_e_), Shannon's information index (*I*), and diversity (*H*).

A total of 43 haplotypes were detected in the three assayed populations. The number of haplotypes per population ranged from 11 to 18, the number of private haplotypes ranged from nine to 16, the effective number of haplotypes ranged from 4.55 to 13.36, the haplotypic richness ranged from 8.04 to 16.00, and the gene diversity ranged from 0.81 to 0.97 (Table 2). The number of alleles per locus ranged from one to eight per population, the effective number of alleles ranged from 1.00 to 3.90, Shannon's information index ranged from 0.00 to 1.52, and the diversity ranged from 0.00 to 0.74 (Table 3). The 10 polymorphic loci could also be successfully amplified in five individuals in each of the following four related species: *Cryptomeria japonica* var. *sinensis*,* Taxodium distichum*,* T. ascendens*, and *Cunninghamia lanceolata* (Table 4, Appendix 1).

**Table 2:** Haplotype diversity in three Chinese *Glyptostrobus pensilis* populations based on 10 polymorphic chloroplast microsatellite markers.^a^

Population	*A*	*P*	*N*_e_	*R*_h_	*H*_e_	*D* ^2^_sh_
DM (*N* = 33)	18	16	7.026	11.857	0.884	76.6
GZHN (*N* = 21)	17	15	13.364	16.000	0.971	470.5
PNSL (*N* = 29)	11	9	4.546	8.035	0.808	1.4
Mean	15.333	13.333	8.312	11.964	0.888	182.8

Note

*A* = number of haplotypes; *P* = number of private haplotypes; *N*_e_ = effective number of haplotypes; *R*_h_ = haplotypic richness; *H*_e_ = genetic diversity; *D* ^2^_sh_ = mean genetic distance between individuals; *N* = number of individuals sampled.

a Locality and voucher information are provided in Appendix 1.

**Table 3:** Characteristics of 10 polymorphic chloroplast microsatellite markers in 83 individuals of three Chinese *Glyptostrobus pensilis* populations.^a^

Locus	DM (*N* = 33)				GZHN (*N* = 21)				PNSL (*N* = 29)			
	*N*_a_	*N*_e_	*I*	*H*	*N*_a_	*N*_e_	*I*	*H*	*N*_a_	*N*_e_	*I*	*H*
Gp_cp_1	3	1.824	0.765	0.452	2	1.960	0.683	0.490	2	1.890	0.664	0.471
Gp_cp_6	2	1.198	0.305	0.165	3	2.194	0.852	0.544	3	1.324	0.479	0.245
Gp_cp_7	3	2.139	0.883	0.533	3	2.845	1.071	0.649	3	1.979	0.779	0.495
Gp_cp_8	3	1.280	0.437	0.219	3	2.110	0.832	0.526	1	1.000	0.000	0.000
Gp_cp_11	5	1.806	0.917	0.446	5	3.084	1.301	0.676	1	1.000	0.000	0.000
Gp_cp_12	3	1.280	0.437	0.219	2	1.893	0.665	0.472	1	1.000	0.000	0.000
Gp_cp_13	8	2.515	1.371	0.602	6	3.903	1.524	0.744	3	1.235	0.398	0.190
Gp_cp_14	3	1.203	0.363	0.169	3	2.110	0.832	0.526	1	1.000	0.000	0.000
Gp_cp_17	2	1.424	0.474	0.298	3	2.384	0.940	0.580	2	1.071	0.150	0.067
Gp_cp_35	2	1.271	0.369	0.213	2	1.995	0.692	0.499	1	1.000	0.000	0.000
Mean	3.400	1.594	0.632	0.331	3.200	2.448	0.939	0.571	1.800	1.250	0.247	0.147

Note

*N* = number of individuals sampled; *N*_a_ = number of alleles; *N*_e_ = effective number of alleles; *I* = Shannon's information index; *H* = diversity.

a Locality and voucher information are provided in Appendix 1.

**Table 4:** Results of cross‐amplification of 10 polymorphic chloroplast microsatellite markers developed for *Glyptostrobus pensilis* in four closely related species.^a^^,^ b

Locus	*Taxodium distichum* (*N* = 5)	*Taxodium ascendens* (*N* = 5)	*Cryptomeria japonica* var. *sinensis* (*N* = 5)	*Cunninghamia lanceolata* (*N* = 5)
Gp_cp_1	254–256	250–254	258–262	254–256
Gp_cp_6	179	179	197	171
Gp_cp_7	149	149	133	177
Gp_cp_8	276	276	284	286
Gp_cp_11	379–383	381–383	375	371
Gp_cp_12	378	378	384	432
Gp_cp_13	303–305	303	299	299
Gp_cp_14	205	205	201–213	213
Gp_cp_17	117	115–117	117	117
Gp_cp_35	116–117	114–117	115–116	116–117

Note

*N* = number of individuals sampled.

a Locality and voucher information are provided in Appendix 1.

b Numbers shown represent the size in base pairs of the amplified fragments.

## CONCLUSIONS

In this study, we developed 10 polymorphic cpSSRs (as well as 55 pairs of monomorphic primers) that can be used to assess the population genetic and phylogeographic structure of *G. pensilis* populations. The high number of private haplotypes in the three assayed populations suggests geographically isolated populations. Additionally, the 10 loci can be successfully amplified in four related species of *G. pensilis*.

## Data Availability

All polymorphic primer sequences were uploaded to the National Center for Biotechology Information (accession number: MK386658–MK386667; Table 1).

## APPENDIX 1 Sampling information for species in this study. All voucher specimens are deposited at the herbarium of Central South University of Forestry and Technology, Changsha, Hunan, China.

**Table:**

Species	Population code	Voucher no.	Collection locality	Geographic coordinates	Elevation (m)	*N*
*Glyptostrobus pensilis* (Staunton ex D. Don) K. Koch	PNSL	Lin170804	Pingnan, Fujian, China	27°0′27.87″N, 118°51′59.75″E	1260	29
	GZHN	Lin170411	Guangzhou, Guangdong, China	23°11′24.6″N, 113°21′38.13″E	40	21
	DM	Lin170729	Doumen, Guangdong, China	22°23′42.5″N, 113°15′14.65″E	20	33
*Taxodium distichum* (L.) Rich.		Li180522	Changsha, Hunan, China	28°8′16.48″N, 112°59′28.36″E	90	5
*Taxodium ascendens* Brongn.		Li180522	Changsha, Hunan, China	28°8′16.48″N, 112°59′28.36″E	90	5
*Cryptomeria japonica* (Thunb. ex L. f.) D. Don var. *sinensis* Miq.		Wang180720	Jiujiang, Jiangxi, China	29°32′59.77″N, 115°58′03.32″E	911	5
*Cunninghamia lanceolata* (Lamb.) Hook.		Li180522	Changsha, Hunan, China	28°8′16.48″N, 112°59′28.36″E	90	5

Note

*N* = number of individuals sampled.

## APPENDIX 2 Characteristics of 55 monomorphic chloroplast microsatellite primers developed in *Glyptostrobus pensilis*.

**Table:**

Locus	Primer sequences (5′–3′)	Repeat motif	Product size (bp)
Gp_cp_2	F: ACATTGATTTCTAAAAGAGAGGAGTCA	(A)_11_	211
	R: TCAGTGTCAGAAATTTGGCTGA		
Gp_cp_3	F: TGATGAGCTACTCTACGTGCT	(T)_13_	369
	R: ATCTGCCATTGTACCCGCAA		
Gp_cp_4	F: ATAGATTCCGAGCGGCTGTG	(T)_18_	292
	R: ACCGCTGAGTTATATCCCTTTCC		
Gp_cp_5	F: GCGATCGTACCTTCATCGGA	(T)_20_	230
	R: TCCTTTTTCAATATCGTTCCCTGG		
Gp_cp_9	F: ATTTCTCGCCAAGCTGTCCA	(AT)_10_	332
	R: CGAGCAATGCCATCTCCTACT		
Gp_cp_10	F: CGAACCCGCATCGTTAGCTT	(A)_15_	280
	R: GGTTGTTCACCTGAAATTAAGAGGA		
Gp_cp_15	F: TCAAGCAAAGGTAGATGGTGAG	(A)_12_	257
	R: TCTCAACCTTCATGTGGGAG		
Gp_cp_16	F: ATGCTCTTTCGCAACGTTCG	(A)_12_	201
	R: TGAACACAAAGAAAGGTAAGGTCT		
Gp_cp_18	F: TCCGCTCAATTCCGTTACTC	(T)_12_	145
	R: TCCATGATTGATTTTCCCTTCGT		
Gp_cp_19	F: TCTTGCAAAATCCGGACCG	(A)_11_	201
	R: TGAACCAAGTCAGTTCGCTTG		
Gp_cp_20	F: CGAAAACCGTCGGGAAACAT	(A)_11_	260
	R: GCTTCTTCCTTCCCGCCAT		
Gp_cp_21	F: GGCTCGCGGGTATGTTAACT	(A)_11_	190
	R: TCGGGCAATTTTGTCATGTACC		
Gp_cp_22	F: AGGGGCAGAATCTAGGGTT	(A)_11_	194
	R: CCGCTATTTTCCACGTTGAGC		
Gp_cp_23	F: ATCCGCCTTGATTCCCGTTT	(A)_11_	263
	R: ACAGGCGCTGTGGAAAGAT		
Gp_cp_24	F: TCTCTTTTGCGTCCTTCCCC	(T)_11_	231
	R: AAGAATTAGTTCGCCATGGGT		
Gp_cp_25	F: TCCTTCGGGATTAATTCTTCATTCT	(T)_11_	264
	R: AATCCTGAGCAGCCAAACC		
Gp_cp_26	F: TTGTAGCTCTACGTGGCAC	(AT)_11_	263
	R: AGGCATAAACAAAAACAGGGCT		
Gp_cp_27	F: CGGGGGAATGATACCTGTCG	(T)_10_	138
	R: ACGGAGACTTGATATTGATGCTC		
Gp_cp_28	F: TCGTGAATTCGTTGGACAG	(TA)_10_	213
	R: TCCATCTGACTCACTCGACT		
Gp_cp_29	F: GAGCTTACTTGGGTACTGAGC	(A)_10_	126
	R: CATCCGGCTCGAGCAATAGT		
Gp_cp_30	F: TGAGTATCCGTTTCCTTTCTTTTGC	(A)_10_	201
	R: TAAGTTTTCCCTTACTATAGTGTGTGT		
Gp_cp_31	F: CGGGAAGAGTAGTATGAAGCTC	(A)_10_	233
	R: GCATATGTGCGATGAATAGACTCC		
Gp_cp_32	F: CCGAGAACGAACCGAATGGA	(A)_10_	157
	R: GGGATTGACTGTTGGATTGGC		
Gp_cp_33	F: ATTAGCGGGGAGTTCCATCC	(A)_10_	226
	R: CGGACTTGTGATTCGTTTGATCT		
Gp_cp_34	F: ACGCGGCGATCAATTGGATA	(A)_10_	143
	R: CCTACAGAGCGTGATCCTGC		
Gp_cp_36	F: TCATTTTTACCCAGGAATAGAAACAT	(A)_10_	156
	R: GATGGCTTCATTTTATTCATAGTTTGT		
Gp_cp_37	F: ACCCAAAAAGAGGAGACAAGC	(A)_10_	165
	R: GAATGACTTCGGGGTGGGAG		
Gp_cp_38	F: ACTTGGACGAACTCCCTATTGA	(AT)_9_	221
	R: CAGCCGGGATAGCTCAGTTG		
Gp_cp_39	F: TCTTATGTTCTTAGTAACACGCCT	(A)_9_	125
	R: TGGAGTAGGAGGAAAATCCGT		
Gp_cp_40	F: ATGTCTCGTTATCGCGGACC	(A)_9_	248
	R: TGACCTGTTGATCCCTTGGC		
Gp_cp_41	F: CTGCACATCTGTCCCTCTGT	(T)_9_	162
	R: TGCTTTCATCCTCCCGCAAT		
Gp_cp_42	F: TGCGATCGTAAGGAAATCCA	(A)_9_	211
	R: TTCTCCCCTGAAGCCATTGG		
Gp_cp_43	F: GGTTGATGGCTCTGGTCTTGA	(A)_9_	186
	R: TGAATCCTTGTTGCTCGGCT		
Gp_cp_44	F: CCATTCGATCCCTATCCGGTC	(T)_9_	224
	R: CATCAACCACTCGGCCATCT		
Gp_cp_45	F: AGTGAGGTAGATTACGCCTAATCT	(A)_9_	222
	R: AGCCCAGTGTTCATTTTGAATATT		
Gp_cp_46	F: GCGAGTCAAGCCGAAGTACA	(A)_9_	132
	R: AATTTTTCGTTTCCTTCGTACTACT		
Gp_cp_47	F: GAAGCAACCGCCAATTCTTCA	(T)_9_	190
	R: TGTTCGGGTGAGAAAGGTGT		
Gp_cp_48	F: TCTCTTACATATCTCTGGAAAAAGGA	(T)_9_	228
	R: TGCTGCTCTGTCCCAACTAT		
Gp_cp_49	F: AGCGAAGAATCCCTTGTCCTG	(A)_9_	171
	R: ATCTGGGCCCTCCGTCTAAT		
Gp_cp_50	F: CAGATACTGGCCGGGCTAGA	(A)_9_	140
	R: CGCTCAGCCATCTCTCCTAG		
Gp_cp_51	F: CGCCATCTTGGATGGAATGG	(T)_9_	233
	R: TGTGGCGGGTATAGTTTAGTGG		
Gp_cp_52	F: CGGCTTTTAAGTGCGACTATGG	(A)_9_	298
	R: TGACTTAATCACCCGCACTC		
Gp_cp_53	F: GGCACGAGAACTTGAAGATCG	(A)_9_	164
	R: ATTGATTCATCGACCCGCGG		
Gp_cp_54	F: TGCATAAGAATGAGCCAACTTGA	(T)_9_	187
	R: TCATACGGCTTAAACAAGAACAC		
Gp_cp_55	F: CAGGCATTTACTTTTTGTTTTGGAGT	(T)_9_	134
	R: TTTGGGTGGAATGGGGATTG		
Gp_cp_56	F: ATATTCCGCAAGAATTTTGGGTT	(T)_9_	202
	R: TGCATTTGTCAACTTGTTTATCGAGA		
Gp_cp_57	F: CGCACGGCTCCTAAGTGAT	(T)_9_	257
	R: ACCCTAAGATGAGCATCGC		
Gp_cp_58	F: TGTGTATTTGGCTTTGAAACGA	(T)_9_	176
	R: TGTCTTTGTTTGCTCAATTTTGC		
Gp_cp_59	F: TATTGGACCAGCGGTAGTGG	(T)_9_	144
	R: ATAAGCAGTCCAAGGGGAGC		
Gp_cp_60	F: ACGATTATTCAGATTGAGCTCCGA	(T)_9_	201
	R: CCCCATTTACCTGTATGCTATACT		
Gp_cp_61	F: GTTCAGCCAATAGGGGAGGG	(T)_9_	159
	R: TAAGTCCCAGGTCCCGCAT		
Gp_cp_62	F: TGTCTACGTGCATAAACTCTTTTC	(T)_9_	209
	R: ACCACGCTCATCTCATGTAC		
Gp_cp_63	F: CCACCTATGCCCATACGGTC	(T)_9_	127
	R: TCGATTGACCTGAGGACCTT		
Gp_cp_64	F: GGGTACCGGGTTCTATTGAAT	(A)_8_	149
	R: TCGATCTATGCCGCCTTACT		
Gp_cp_70	F: TCGAGCCGTATGAAGATAAACCT	(G)_11_	137
	R: GCTCTTCCTTCGCTTCGAG		

Optimal annealing temperature was 55°C for all loci.

## References

[aps311277-bib-0001] Averyanov, L. V. , K. L. Phan , T. H. Nguyen , S. K. Nguyen , T. V. Nguyen , and T. D. Pham . 2009 Preliminary observation of native *Glyptostrobus pensilis* (Taxodiaceae) stands in Vietnam. Taiwania 54: 191–212.

[aps311277-bib-0002] Eliades, N. G. , and D. G. Eliades . 2009 HAPLOTYPE ANALYSIS: Software for analysis of haplotypes data. Forest Genetics and Forest Tree Breeding, Georg‐August University, Goettingen, Germany.

[aps311277-bib-0003] Ennos, R. A. , W. T. Sinclair , X. S. Hu , and A. Langdon . 1999 Using organelle markers to elucidate the history, ecology and evolution of plant populations *In* Hollingsworth P. M., Bateman R. M., and Gornall R. J. [eds.], Molecular systematics and plant evolution. Taylor & Francis, London, United Kingdom.

[aps311277-bib-0004] Gryta, H. , C. Van de Paer , S. Manzi , H. Holota , M. Roy , and G. Besnard . 2017 Genome skimming and plastid microsatellite profiling of alder trees (*Alnus spp*., Betulaceae): Phylogenetic and phylogeographical prospects. Tree Genetics and Genomes 13: 118.

[aps311277-bib-0005] Hao, Z. D. , T. L. Cheng , R. H. Zheng , H. B. Xu , Y. W. Zhou , M. P. Li , F. J. Lu , et al. 2016 The complete chloroplast genome sequence of a relict conifer *Glyptostrobus pensilis*: Comparative analysis and insights into dynamics of chloroplast genome rearrangement in Cupressophytes and Pinaceae. PLoS ONE 11: e0161809. 27560965 10.1371/journal.pone.0161809 PMC4999192

[aps311277-bib-0006] Hulce, D. , X. Li , T. Snyder‐Leiby , and C. S. J. Liu . 2011 GeneMarker genotyping software: Tools to increase the statistical power of DNA fragment analysis. Journal of Biomolecular Techniques 22(Suppl): S35–S36.

[aps311277-bib-0007] Li, B. , and H. X. Liu . 2012 Research advances in chloroplast simple sequence repeat (cpSSR). Journal of Anhui Agricultural Sciences 40: 7638–7639, 7649.

[aps311277-bib-0008] Li, F. G. , and N. H. Xia . 2004 The geographical distribution and cause of threat to *Glyptostrobus pensilis* (Taxodiaceae). Journal of Tropical and Subtropical Botany 12: 13–20.

[aps311277-bib-0009] Li, F. G. , and N. H. Xia . 2005 Population structure and genetic diversity of an endangered species, *Glyptostrobus pensilis* (Cupressaceae). Botanical Bulletin of Academia Sinica 46: 155–162.

[aps311277-bib-0010] Nguyen, M. T. , D. D. Vu , T. T. X. Bui , and M. D. Nguyen . 2013 Genetic variation and population structure in Chinese water pine (*Glyptostrobus pensilis*): A threatened species. Indian Journal of Biotechnology 12: 499–503.

[aps311277-bib-0011] Peakall, R. , and P. E. Smouse . 2012 GenAlEx version 6.5: Genetic analysis in Excel. Population genetic software for teaching and research–An update. Bioinformatics 28: 2537–2539. 22820204 10.1093/bioinformatics/bts460 PMC3463245

[aps311277-bib-0012] Pleines, T. , S. S. Jakob , and F. R. Blattner . 2009 Application of non‐coding DNA regions in intraspecific analyses. Plant Systematics and Evolution 282: 281–294.

[aps311277-bib-0013] Ruhsam, M. , A. Clark , A. Finger , A. S. Wulff , R. R. Mill , P. I. Thomas , M. F. Gardner , et al. 2016 Hidden in plain view: Cryptic diversity in the emblematic *Araucaria* of New Caledonia. American Journal of Botany 103: 888–898. 27208357 10.3732/ajb.1500487

[aps311277-bib-0014] Thomas, P. , and B. A. LePage . 2011 The end of an era?: The conservation status of redwoods and other members of the former Taxodiaceae in the 21st century. Japanese Journal of Historical Botany 19: 89–100.

[aps311277-bib-0015] Thomas, P. , Y. Yang , A. Farjon , D. Nguyen , and W. Liao . 2011 Glyptostrobus pensilis. The IUCN Red List of Threatened Species 2011: e.T32312A9695181. 10.2305/iucn.uk.2011-2.rlts.t32312a9695181.en [accessed 10 November 2011].

[aps311277-bib-0016] Tsumura, Y. , K. Yoshimura , N. Tomaru , and K. Ohba . 1995 Molecular phylogeny of conifers using RFLP analysis of PCR‐amplified specific chloroplast genes. Theoretical and Applied Genetics 91: 1222–1236. 24170050 10.1007/BF00220933

[aps311277-bib-0017] Ueno, S. , Y. Moriguchi , K. Uchiyama , T. Ujino‐Ihara , N. Futamura , T. Sakurai , K. Shinohara , and Y. Tsumura . 2012 A second generation framework for the analysis of microsatellites in expressed sequence tags and the development of EST‐SSR markers for a conifer, *Cryptomeria japonica* . BMC Genomics 13: 136. 22507374 10.1186/1471-2164-13-136 PMC3424129

[aps311277-bib-0018] Untergasser, A. , H. Nijveen , X. Y. Rao , T. Bisseling , R. Geurts , and J. A. M. Leunissen . 2007 Primer3Plus, an enhanced web interface to Primer3. Nucleic Acids Research 35: W71–W74. 17485472 10.1093/nar/gkm306 PMC1933133

[aps311277-bib-0019] Vendramin, G. G. , L. Lelli , P. Rossi , and M. Morgante . 1996 A set of primers for the amplification of 20 chloroplast microsatellites in Pinaceae. Molecular Ecology 5: 595–598. 8794566 10.1111/j.1365-294x.1996.tb00353.x

[aps311277-bib-0020] Wang, G. T. , Z. F. Wang , R. J. Wang , D. Liang , and G. B. Jiang . 2019 Development of microsatellite markers for a monotypic and globally endangered species, *Glyptostrobus pensilis* (Cupressaceae). Applications in Plant Sciences 7(2): e1217. 10.1002/aps3.1217 PMC6384295 30828504

[aps311277-bib-0021] Wu, Z. Y. 2011 Study on conservation biology and restoration technique of the relict plant Glyptostrobus pensilis. Ph.D. dissertation, Fujian Agriculture and Forestry University, Fujian Province, China.

